# Exploring the Increased Activity of the Blue Light-Dependent Photoenzyme Fatty Acid Photodecarboxylase under Violet Light

**DOI:** 10.1021/acscatal.4c07757

**Published:** 2025-03-31

**Authors:** Harry J. Spacey, Daniel Healy, Jason M. D. Kalapothakis, Junfeng Ma, Michiyo Sakuma, Perdita E. Barran, Derren J. Heyes, Nigel S. Scrutton

**Affiliations:** Manchester Institute of Biotechnology, University of Manchester, 131 Princess Street, Manchester M1 7DN, U.K.

**Keywords:** flavin, photoenzyme, excited state, violet light, fatty acid photodecarboxylase

## Abstract

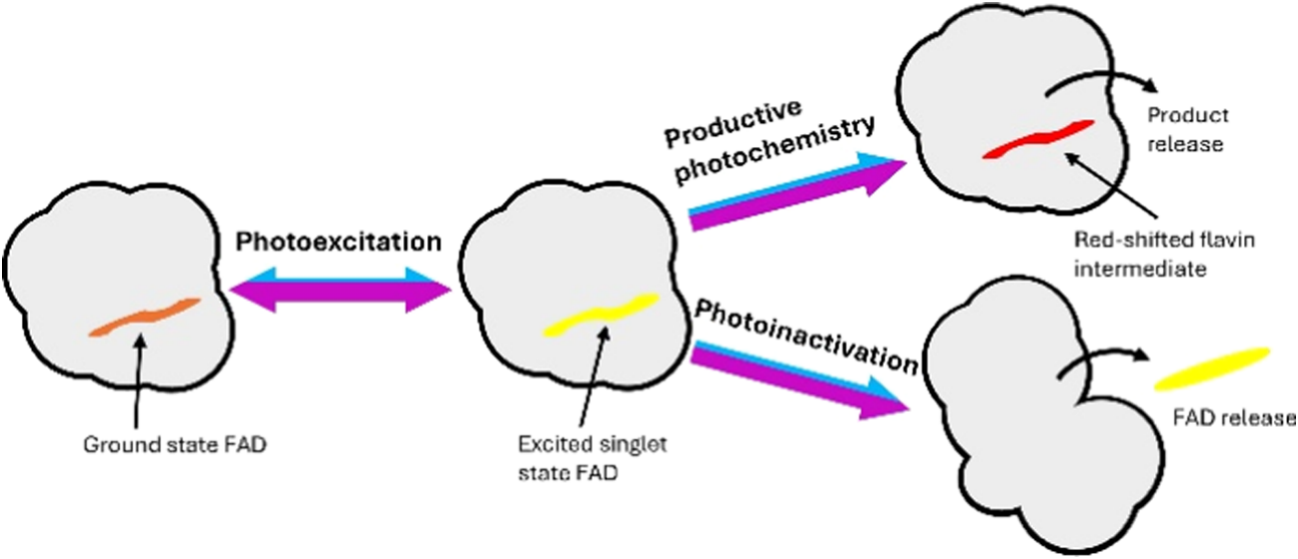

Fatty acid photodecarboxylase (FAP) is a FAD-containing enzyme that catalyzes the light-driven decarboxylation of medium-long chain fatty acids. Over recent years, the substrate scope of FAP has been broadened to improve its potential to catalyze a range of blue light-dependent industrially relevant reactions. However, photoinactivation constitutes a major hurdle for generalized applications. Previous studies have suggested that violet light may be a more suitable illumination wavelength for many of these applications. Here, we have investigated any possible enhancement in the catalytic activity of FAP upon illumination with violet light and utilized a spectrophotometric assay that detects the production of CO_2_ in real time to monitor the FAP reaction under different illumination conditions. We show that the activity of FAP at low intensities of violet light is approximately 6-fold higher than under identical illumination conditions with blue light. Moreover, the product yield increases further when the light is delivered in a pulsed manner, most likely as a result of lower levels of photoinactivation than is observed upon continuous illumination. More detailed spectrophotometric measurements have confirmed that FAP employs a similar catalytic cycle upon illumination with both violet and blue light. Rather, the enhancement in catalytic efficiency observed with violet light is attributable to higher populations of excited state FAD species that can proceed along a productive catalytic pathway. We suggest that pulses of low-intensity violet light provide an optimized route for FAP catalysis, highlighting the importance of illumination conditions in the expanding field of flavin-based photobiocatalysis.

## Introduction

Light-driven enzymatic reactions provide a versatile and chemically diverse approach to the production of chemicals and fuels. However, photoenzymes are rarely found in nature.^1^ Fatty acid photodecarboxylase from *Chlorella variabilis* (*Cv*FAP) is the most recently discovered naturally occurring photoenzyme, the only other two being DNA photolyase and protochlorophyllide oxidoreductase.^2−4^*Cv*FAP contains an FAD cofactor in its active site that initiates radical-based photodecarboxylation of medium-long chain fatty acids into their respective *n*-alkene/*n*-alkane products.^4^ The abundant availability of fatty acid substrates and the utility of the products means that *Cv*FAP could become an essential enzyme in the production of biofuels and other hydrocarbons.

Photoenzymes are able to catalyze high-energy reactions that are not possible with thermally activated enzymes. Upon blue light illumination, the FAD cofactor in FAP is excited to the singlet state and abstracts an electron from the carboxyl group of the fatty acid substrate to form a carboxyl radical and an FAD semiquinone. The carboxyl radical then rapidly decarboxylates, releasing CO_2_ into solution, primarily in the form of HCO_3_^–^.^5^ Subsequent electron and proton transfer steps, possibly involving active site cysteine or arginine residues^5,6^ convert the resulting alkyl radical into the alkane product. Reduction of the alkyl radical is coupled with the reoxidation of the FAD semiquinone, via an unusual red-shifted flavin intermediate, to complete the catalytic cycle.

The potential of FAP as a versatile biocatalyst for industrial applications has been augmented by the creation of numerous variants of *Cv*FAP, broadening the substrate scope of this photoenzyme.^7−9^ These include variants that can accommodate shorter-chain fatty acids in the binding pocket resulting in improved turnover of C_3_–C_14_ substrates and providing a promising route to green bioalkane production.^10−12^ More recently, Ju and colleagues have expanded the substrate scope of FAP further through directed evolution approaches to produce FAP variants capable of stereoselective photocyclization by forming C–C bonds.^13^ Similar photocyclization reactions have also been performed using repurposed thermally activated flavin-containing ‘ene’ reductases (EREDs).^14,15^ The EREDs have also been modified to catalyze a range of different light-driven reactions,^16−19^ highlighting the huge potential of flavin-dependent enzymes in the emerging field of photobiocatalysis.

In the majority of cases, blue light has been utilized in photobiocatalysis to drive reactions, due to the strong absorbance of the flavin cofactor in this region of the visible spectrum. Recently, there has been an increased focus on illumination conditions to enhance the efficiency of photoenzymatic reactions. Spectral tuning of EREDs to favor lower-energy red light excitation has demonstrated the potential to enhance the specificity and stability of light-driven enzymes in large-scale photobiocatalysis.^20^ Furthermore, recent studies have suggested that illumination of *Cv*FAP with violet light (∼395 nm) can result in more efficient decarboxylation of the palmitic acid substrate compared to blue light.^21^ Bréda and colleagues showed higher product formation (99% conversion in just 4 min) upon illumination with lower-power violet light (50 W violet LED) rather than standard blue light irradiation (79% conversion with a 300 W blue LED in 60 min). However, the disparity in the intensity of the illumination conditions used, coupled with the current lack of a robust continuous assay for measuring real-time kinetic activity of the FAP reaction, means that it is difficult to fully understand the extent to which violet light does or does not enhance the activity of FAP. Until now, kinetic data for FAP activity have only been measured using time-point assays that require laborious chromatographic separation methods,^22^ thus preventing any detailed kinetic analysis. A spectroscopic method for monitoring photodecarboxylation is currently missing from the available toolkit used to study FAP catalysis. The development of such an assay would be necessary to discern any differences in FAP activity upon illumination with different wavelengths of light. Here, we have used a combination of approaches to explore the enhancement in FAP activity upon illumination with violet light compared to blue light, including the implementation of a stopped-flow time-resolved spectrophotometric assay for tracking the release of CO_2_/HCO_3_^–^ that allows FAP activity to be monitored in real-time. Further biophysical approaches and kinetic modeling have provided a mechanistic rationale for the enhancement in the catalytic activity of FAP under violet light illumination and highlight the importance of optimizing illumination conditions for photobiocatalysis by FAP and other flavin-based photoenzymes.

## Materials and Methods

### Materials

All materials were of analytical grade and were purchased from Sigma-Aldrich unless otherwise stated.

### Expression and Purification of *Cv*FAP

A truncated, codon-optimized *Cv*FAP sequence (residues 76–654) was synthesized (Geneart, Life Technologies) and cloned into a pET21a (C-terminal histidine-tagged thioredoxin). All expression and purification steps were performed in the dark or under a low-power red light. The plasmid was transformed into BL21 (DE3) *Escherichia coli* cells for expression. Cells were grown in 500 mL of terrific broth (TB) media at 37 °C and 180 rpm agitation in 2 L flasks to an OD_600_ of 0.8 before induction with 0.2 mM IPTG. The temperature was reduced to 18 °C and the cells were grown for 18 h at 180 rpm. Cells were harvested by centrifugation at 4 °C, 5000 rpm for 20 min and the pellet was stored at −80 °C.

All purification processes were performed at 4 °C. Cell pellets were thawed and resuspended in 50 mM Tris (pH 8.5), 300 mM NaCl, 5% glycerol, 0.25 mg mL^–1^ lysozyme and 10 μg mL^–1^ DNase. Protease inhibitor cocktail tablets were added to the suspension to prevent proteolysis. Cells were lysed by sonication and centrifuged at 18,000*g* for 1 h. The supernatant was loaded onto a nickel-charged column which had been pre-equilibrated with Buffer A (50 mM Tris (pH 8), 300 mM NaCl, and 5% glycerol). The column was washed with Buffer B (50 mM Tris (pH 8), 300 mM NaCl, 5% glycerol and 2 mM imidazole) to remove nonspecifically bound proteins. A final elution step was performed with Buffer C (50 mM Tris (pH 8), 300 mM NaCl, 5% glycerol and 250 mM imidazole). Purified *Cv*FAP was desalted by gel filtration using a Superdex 200 26/600 mm column (GE HealthCare) and frozen in liquid nitrogen before being stored at −80 °C. The concentration of *Cv*FAP was measured with 1 cm path length quartz cuvettes in a Cary 60 UV–vis spectrophotometer (Agilent Technologies) using the extinction coefficients 63.3 at 280 nm and 11.3 mM^–1^ cm^–1^ at 469 nm.^4^

### Coupled Assay to Monitor CO_2_/Bicarbonate Formation

All reactions using the enzyme-coupled assay were performed on a stopped-flow spectrometer (Applied Photophysics Ltd.) in an anaerobic glovebox (Belle Technology) to prevent interference from atmospheric CO_2_ during the reaction. Buffers (Tris-HCl 100 mM pH 8) were filtered then degassed by bubbling with nitrogen for 1–2 h and left in the glovebox overnight to allow all bicarbonate to diffuse out of solution as CO_2_ as described by Moody and colleagues.^23^ The final assay mix used in the reaction cell is shown in Table S1. The full assay mix was made up 10 min before the experiment to allow any trace amounts of bicarbonate to be removed prior to performing the assay. For assays with FAP, the assay mix was added to both syringes, with one syringe containing the FAP enzyme and one syringe containing the palmitic acid. Thirty % DMSO was used to dissolve the palmitic acid and vortexed extensively until it was fully in solution. The stopped-flow experiments were run at 25 °C. Illumination directly into the reaction cell at the stated wavelengths and light intensity was provided by high-power LEDs (Thorlabs, emission of each of the LEDs is shown in Figure S1). The development of the assay is described in the results section but involved monitoring the consumption of NADH at 340 nm during the reaction. A 343 nm bandpass filter (Thorlabs) was placed before the detector to prevent any light contamination from the external LED used to trigger the FAP reaction. The stopped-flow device was washed with degassed buffer to remove any bicarbonate from the internal environment prior to any measurements. To avoid inactivation of *Cv*FAP, loading steps were performed under a low-power red lamp and reactions were run in triplicate for each condition tested

Under standard aerobic conditions, there was significant background activity from CO_2_ in the air dissolved into the buffer during the reaction (Figure S2). To prevent atmospheric CO_2_ from interfering with the reaction, the assay was conducted in an N_2_-containing glovebox to ensure a CO_2_-free environment. The addition of 30% DMSO to the buffer increased the enzyme rate 10-fold, which was crucial for processing bicarbonate released by *Cv*FAP at a sufficient rate to avoid any buildup of bicarbonate (Figures S3 and S4). DMSO was necessary to dissolve palmitic acid in the Tris buffer. In addition, the intensity of the 340 nm probe light was reduced to minimize background FAP activity (Figure S5).

### Product Determination Using Flame-Ionized Detection Gas Chromatography

Following enzyme induction (using the protocol above), cells were harvested by centrifugation at 1000*g* at 4 °C for 15 min and washed with 100 mM Tris-HCl/NaCl buffer at pH 8. Cells were resuspended in the same buffer and protein concentration was determined using standardized protein concentration on SDS-Page with ImageStudio Gel imaging software. One mL of the resuspended cell pellet was combined with 200 μM palmitic acid and the reaction was initiated using a 395 or 455 nm LED and quenched by placing samples in the dark. Reactions were carried out in a 3 mL glass vial at 180 rpm at 30 °C in an AlgaeTron incubator (Photon Systems Instruments). Pentadecane production was measured using gas chromatography with flame-ionized detection (GC-FID) with an HP-1 column (Agilent Technologies). These samples were mixed with ethyl acetate and 0.1% (v/v) *sec*-butyl benzene. Samples were centrifuged for 1 min using a benchtop centrifuge. MgCl_2_ was added to the supernatant and centrifuged for a further 2 min. The supernatant was subsequently removed and analyzed.

### Activity Measurements Using Continuous and Pulsed Illumination

The product yield from FAP-catalyzed reactions was determined by illuminating samples in a quartz cuvette with mounted 395 or 455 nm LEDs at 100 μmol photons m^–2^ s^–1^. Reaction conditions were 3 μM *Cv*FAP with 500 μM palmitic acid in 70 mM Tris-HCl buffer containing 30% DMSO at pH 8. Illumination was performed in both a continuous manner and by using 100 ms pulses from the LEDs and mediated by a TGP110 pulse generator (Thurlby Thandar) where the period between pulses was varied. Reactions were performed in triplicate and were run for a time that equated to 1 min of continuous photon delivery. The product was then extracted in ethyl acetate for analysis of pentadecane formation using gas chromatography (as described above).

### Fluorescence Spectroscopy

Fluorescence experiments were performed in a stopped-flow device (TgK Scientific) under aerobic conditions. Reaction conditions were 10 μM *Cv*FAP with/without 500 μM palmitic acid in 70 mM Tris-HCl buffer containing 30% DMSO at pH 8. LEDs were mounted on a clamp stand that allowed illumination of the reaction cell through a window in the stopped-flow device. The pulse width was 100 ms with a 900 ms period between pulses, resulting in 1 pulse per second over 60 s. Fluorescence was measured from each LED pulse by using a photomultiplier tube detector fitted with a 500 nm long-pass filter. Fluorescence emission was measured as a % of total fluorescence of 10 μM FMN following illumination with violet light at 100 μmol photons m^–2^ s^–1^.

### Laser Photoexcitation Measurements

Time-gated fluorescence emission spectra were acquired using an image-intensified CCD camera (Andor Technologies) of an LP980 laser flash photolysis instrument (Edinburgh Instruments Ltd.) upon excitation with a laser pulse (6–8 ns) from a Q-switched Nd:YAG laser (NT432, EKSPLA) in a cuvette of 1 cm path length. Emission spectra were recorded over 100 ns between 300 and 700 nm after excitation of a laser pulse (∼2 mJ) at either 395 or 455 nm. Samples contained 25 μM *Cv*FAP in the presence and absence of 500 μM palmitic acid in 70 mM Tris-HCl buffer containing 30% DMSO at pH 8. Spectra shown are the average from 10 laser pulses and the sample was mixed by inverting the cuvette several times between each flash. The total fluorescence emission was obtained by integrating the area under the curve of emission between 500–750 nm.

Detection of the red-shifted flavin intermediate was performed using the same nanosecond laser flash photolysis system. Kinetic absorption transients were recorded at 515 nm with the detection system (comprising probe light, sample, monochromator and photomultiplier) at right angles to the incident laser beam. Samples contained 50 μM *Cv*FAP with 300 μM palmitic acid in 70 mM Tris-HCl buffer containing 30% DMSO at pH 8. The reaction was driven using laser pulses at 355 nm (∼40 mJ) and 455 nm (∼20 mJ) to maximize the signal intensity. Time constants were observed from the average of at least five time-dependent absorption measurements by fitting them to a single exponential function using the L900 software (Edinburgh Instruments Ltd.).

## Results and Discussion

### Increased FAP Activity in a Whole-Cell Environment under Violet Light versus Blue Light

**Figure 1:**
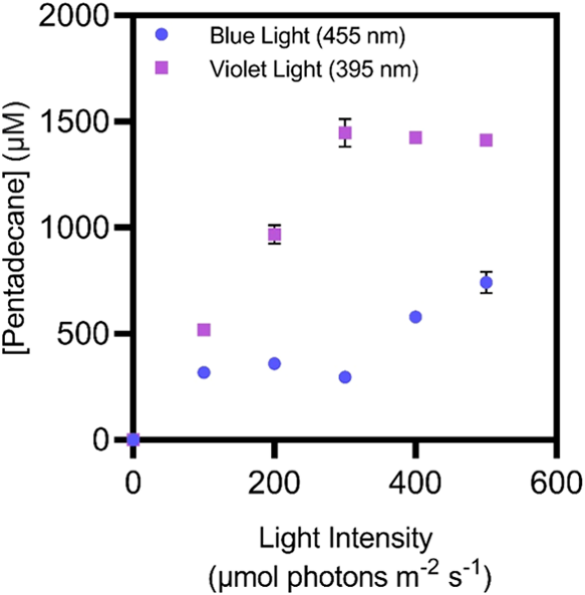
A comparison of pentadecane production after 3 min by varying the photon delivery from the 455 and 395 nm LEDs; light intensities ranged from 0 to 500 μmol photons m^–2^ s^–1^. All reactions were performed at 30 °C using whole-cell *E. coli* overexpressing *Cv*FAP. Reactions were performed in 70 mM Tris-HCl buffer +30% DMSO at pH 8 with 5 mM palmitic acid.

Previous studies have indicated that higher FAP activity was observed upon illumination with violet light compared to blue light.^21^ However, as these measurements were performed under widely varying light intensities^21^ it is unclear whether the enhancement in activity is due to the wavelength of light used or because of an increased number of photons delivered to the sample. Consequently, we have now investigated the activity of *Cv*FAP under blue and violet light in more detail by initially performing assays in *E. coli* under identical light intensities (Figure 1). Whole-cell environments are convenient for screening *Cv*FAP activity, due to higher levels of catalytic activity than in purified enzyme samples, due to less photoinactivation of FAP in whole cells as a result of octanoic acid and other fatty acids occupying the active site at all times.^24,25^ At all light intensities used, *Cv*FAP under violet light demonstrated enhanced catalytic activity.

Under violet light, the amount of pentadecane produced increased sharply up to a light intensity of approximately 300 μmol photons m^–2^ s^–1^ before leveling off at ∼30% conversion yield at higher light intensities. Conversely, under blue light the pentadecane yield increased linearly at all light intensities, reaching a maximum of ∼14% conversion yield at 500 μmol photons m^–2^ s^–1^. Therefore, the photochemical efficiency of FAP is higher with violet light compared to blue light and suggests that lower light intensities can be used with violet light to achieve similar product yields (i.e., lower energy burden), making it an optimal wavelength for alkane production in biotechnological applications.

### A Continuous Assay for Measuring FAP Activity

**Figure 2:**
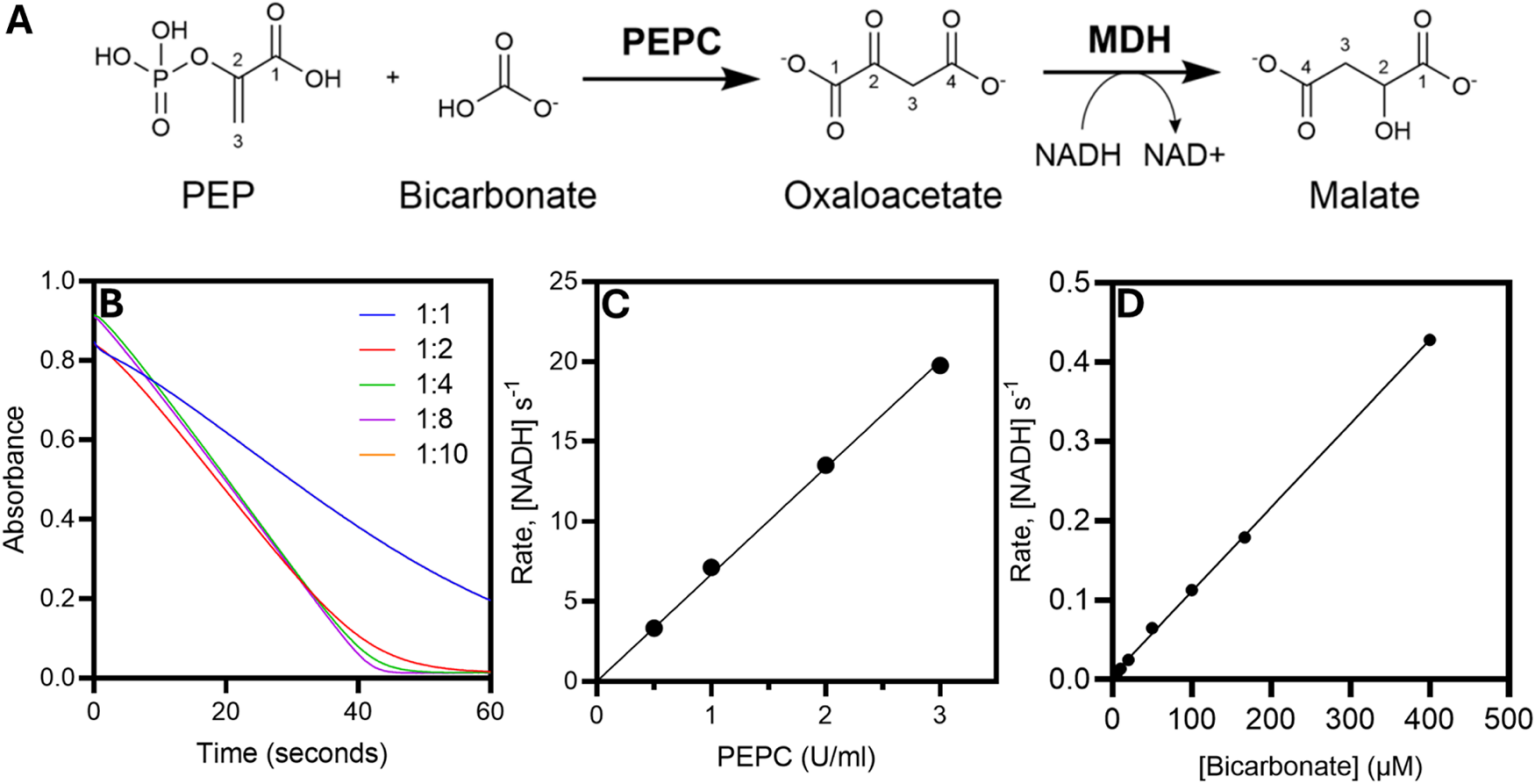
Development of a continuous CO_2_ release assay. (A) Simplified reaction scheme for the coupled-assay. (B) Traces showing NADH consumption at different ratios of PEPC/MDH with an excess (10 mM) of bicarbonate. The enzyme ratios were established by comparing activity units per milliliter (U/ml). The rate of NADH consumption was linearly dependent on (C) PEPC concentration (using a ratio of 1:6 PEPC/MDH) *R*^2^ = 0.99 and (D) bicarbonate concentration *R*^2^ = 0.99. All experiments were performed in Tris-HCl 100 mM at pH 8 at 25 °C.

To investigate the apparent enhancement in the catalytic activity of *Cv*FAP with violet light compared to blue light we sought to develop a continuous assay for monitoring the FAP-catalyzed reaction. The two products from the photodecarboxylation of palmitic acid are pentadecane and CO_2_/HCO_3_^–^. Direct tracking of these products spectrophotometrically is not feasible since they do not absorb light in the UV–visible spectrum. However, studies have shown that HCO_3_^–^ can be assayed by coupling the enzymes phosphoenolpyruvate carboxylase (PEPC) and malate dehydrogenase (MDH) in a linked assay.^23,26−29^ The reaction involves the conversion of HCO_3_^–^ and phosphoenolpyruvate (PEP) into oxaloacetate by PEPC, followed by the conversion of oxaloacetate and NADH into malate and NAD^+^, catalyzed by MDH (Figure 2A). The conversion of NADH to NAD^+^ can be tracked spectrophotometrically by following the decrease in absorbance at 340 nm. This concept has been described previously for determination of dissolved CO_2_/bicarbonate in serum and is available as a commercial kit.^30^ We adapted the usage of this coupled reaction by using PEPC and MDH in sufficient excess to allow direct measurement of continuous CO_2_/HCO_3_^–^ release by FAP. We found that the optimal enzyme composition (expressed as activity units per ml (U/mL)) was a ratio of 1 U/mL PEPC: 6 U/mL MDH. This ratio ensured that the reaction catalyzed by MDH did not become rate-limiting (Figure 2B). We estimated the activity units of PEPC required to measure FAP-mediated HCO_3_^–^ formation by performing assays at varying PEPC (Figure 2C) and bicarbonate concentrations (Figure 2D). There was a linear dependence of the rate of NADH consumption on both PEPC and HCO_3_^–^ concentration. Based on the published kinetic values for *Cv*FAP (*k*_cat_ = 0.31 s^–1^),^22^ we estimated the concentration of PEPC required to consume HCO_3_^–^ at a sufficient rate to track the FAP-catalyzed photodecarboxylation of palmitic acid. As 1 U/mL PEPC and 200 μM bicarbonate yielded a rate of 0.2 μM s^–1^, we rationalized that a final composition of 10 U/mL PEPC and 60 U/mL MDH would ensure that the rate of the linked assay is significantly faster than the rate of FAP catalysis. Further details on conditions used in the assay can be found in the methods section and the Supporting Information.

**Figure 3:**
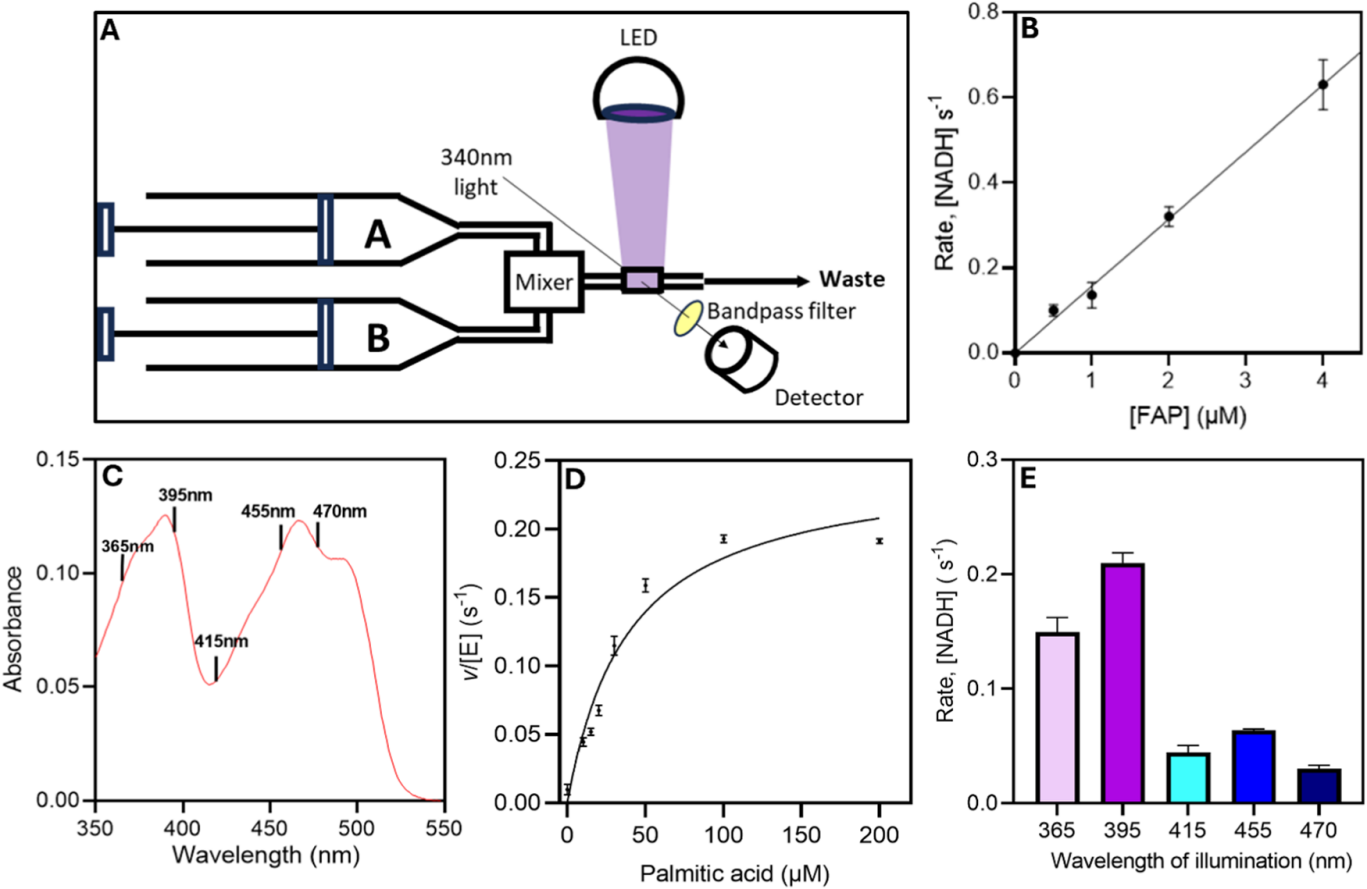
Use of the enzyme-coupled assay to measure *Cv*FAP activity. (A) A schematic representation of the enzyme-coupled assay setup in the stopped-flow device. Syringes A and B were loaded with an assay mix (shown in Table 1) containing either *Cv*FAP (Syringe A) or palmitic acid (Syringe B). Samples were mixed and the reaction cell was exposed to an LED which illuminated the sample, initiating the photodecarboxylation of palmitic acid by *Cv*FAP. The formation of bicarbonate resulted in the loss of NADH through a coupled reaction involving the enzymes MDH + PEPC. NADH consumption was measured spectrophotometrically at 340 nm and rates were determined by taking the gradient of the slope. A bandpass filter was used to prevent any light from the LED from hitting the detector, allowing only the 340 nm light through. (B) The rate of NADH consumption in response to increased *Cv*FAP concentration when illuminated with a 455 nm LED at 1000 μmol photons m^–2^ s^–1^. Fit with linear regression curve *R*^2^ = 0.98. A saturating amount of palmitic acid (200 μM) was used. (C) Absorbance spectrum of *Cv*FAP. The lines indicate the wavelengths used to trigger photocatalysis in the current study. (D) Michaelis–Menten plot of *Cv*FAP decarboxylation activity at increasing concentrations of palmitic acid (*K*_M_ = 39.0 ± 10.4 μM, *k*_cat_ = 0.25 ± 0.03 s^–1^). (E) Rate of NADH consumption upon illumination of *Cv*FAP at 5 different wavelengths of light using an intensity of 3000 μmol photons m^–2^ s^–1^ and 100 μM palmitic acid. Rates were determined by measuring the gradient at the earliest linear region of the traces. The background rate from the assay (when performed in the dark) was subtracted from each measurement to give a final rate. Assays were performed at 25 °C in 70 mM Tris-HCl + 30% DMSO (pH 8), with 1 μM FAP.

The suitability of the assay for the measurement of *Cv*FAP was explored (Figures S1–S5). A stopped-flow device was modified to allow direct illumination of the reaction cell using LEDs at selected wavelengths (Figure 3A). A 340 nm bandpass filter was incorporated prior to the detector to prevent any exogenous light source from influencing the measured spectroscopic changes. To avoid atmospheric CO_2_ dissolving into the buffer, assays were performed inside an anaerobic glovebox and buffers were degassed, meaning any residual bicarbonate (HCO_3_^–^) was depleted by MDH and PEPC prior to the addition of *Cv*FAP. Upon illumination, the rate of NADH consumption was linearly dependent on the concentration of *Cv*FAP, confirming that the photodecarboxylation chemistry is the rate-limiting step in the overall assay (Figure 3B). The value of this assay was further demonstrated by repeating the assay at increasing concentrations of palmitic acid, using 1 μM *Cv*FAP at a light intensity of 1000 μmol photons m^–2^ s^–1^, to determine Michaelis–Menten kinetic parameters. Under these conditions, a *k*_cat_ of 0.25 ± 0.03 s^–1^ and a *K*_m_ for palmitic acid of 39.0 ± 10.4 μM was calculated (Figure 3D), which is similar to the kinetic parameters measured previously using a stopped gas chromatography assay under similar illumination conditions.^22^

The coupled assay was used to monitor the formation of CO_2_/HCO_3_^–^ during the photodecarboxylation of palmitic acid by *Cv*FAP upon illumination with different wavelengths of light (365–470 nm). These wavelengths were chosen to scan across the two main absorption bands of oxidized FAD in *Cv*FAP (Figure 3C). In the blue region of the spectrum, there was only minimal observable difference between 415, 455, and 470 nm illumination, although a slightly higher activity was observed with 455 nm light (Figure 3E). Both violet/ultraviolet wavelengths tested here resulted in higher rates of turnover compared to any of the blue wavelengths, with ∼1.5-fold higher activity for 395 nm compared to 365 nm, presumably reflecting the slightly higher absorbance of the sample at this wavelength. However, there was a ∼3.3-fold higher rate of activity upon illumination with 395 nm light, when compared to 455 nm despite the oxidized FAD absorbing to a similar degree at these two wavelengths.

**Figure 4:**
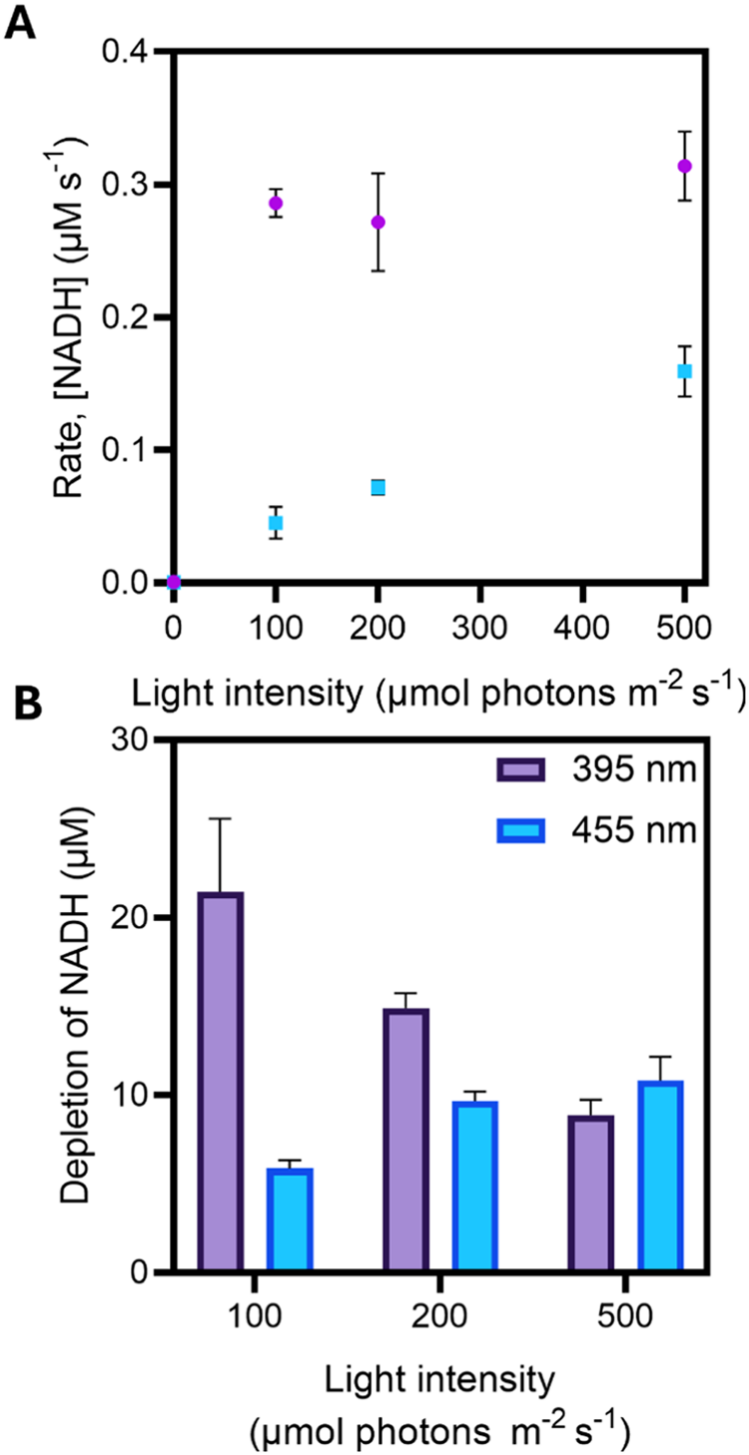
Activity of CvFAP at different light intensities illuminated with 395 nm (violet) and 455 nm (blue) LEDs. (A) Plot of the rate of NADH consumption when illuminating CvFAP and palmitic acid at different light intensities from 0 to 500 μmol photons m^–2^ s^–1^. (B) Total amount of product formed, calculated through NADH depletion over 3 min. Assays were performed at 25 °C in 70 mM Tris-HCl + 30% DMSO (pH 8), with 1 μM FAP and 200 μM palmitic acid.

The dependence of light intensity on the rate of reaction for violet light (395 nm) and blue light (455 nm) reveals significant differences between the two excitation wavelengths (Figure 4A). With blue light, we found a strong linear correlation between light intensity and FAP turnover rate over the range of light intensities studied, which is consistent with previously published findings.^22^ In contrast, the rate of FAP catalysis increases rapidly at low violet light intensity and then reaches a plateau at 100 μmol photons m^–2^ s^–1^. At this light intensity, illumination of FAP with violet light (rate = 0.29 μM s^–1^) exhibited a 6.4-fold increase in the initial rate when compared to blue light (rate = 0.045 μM s^–1^). By measuring the amplitude of the absorbance change after 3 min and dividing by the extinction coefficient of NADH at 340 nm, we were able to obtain an estimate of the total amount of bicarbonate produced during the assay (Figure 4B). With violet light, the production of bicarbonate decreased with light intensity despite having similar initial rates. We reason that this was due to increased photoinactivation from the excess photon delivery. With blue light, the increased light intensity resulted in a greater bicarbonate yield due to the increased rate of catalysis outweighing any increase in photoinactivation.

By performing the assay for longer periods of time to monitor the drop off in enzymatic activity we can estimate the rate of inactivation under different illumination conditions (Figure S6). *Cv*FAP was inactivated much more rapidly upon irradiation with 500 μmol photons m^–2^ s^–1^ violet light (*k*_inact_ = 0.35 min^–1^) compared to the same intensity of blue light (*k*_inact_ = 0.067 min^–1^). Although inactivation also occurs at a faster rate at lower light intensities (100 μmol photons m^–2^ s^–1^) with violet light (*k*_inact_ = 0.11 min^–1^) compared to blue light (*k*_inact_ = 0.012 min^–1^), the substantially faster rate of productive catalysis under these conditions with violet light resulted in greater product yield after 20 min. Previous studies have shown that the formation of oxygen radicals is likely to be involved in the photoinactivation of *Cv*FAP.^31^ Due to the nature of the enzyme-coupled assay, all reactions were performed in the absence of oxygen. As there was still strong light-dependent inactivation of *Cv*FAP, it is unlikely that the formation of oxygen radicals is the prevailing mechanism for light-inactivation of *Cv*FAP in this case. The more likely reason for the observed photoinactivation is photoexcited FAD oxidizing active site residues as suggested by Wu and colleagues.^24^ To the best of our knowledge, aside from FAP no other studies have reported the effects of violet light on flavin-based photobiocatalysis. However, a similar dose-dependent deactivation has been shown for FMN-containing LOV2 domains upon irradiation with violet light, where an activated cysteine-flavin adduct can be broken upon absorption of a near UV photon.^32^

### Mechanistic Insights into the Enhancement in Catalytic Efficiency with Violet Light

**Figure 5:**
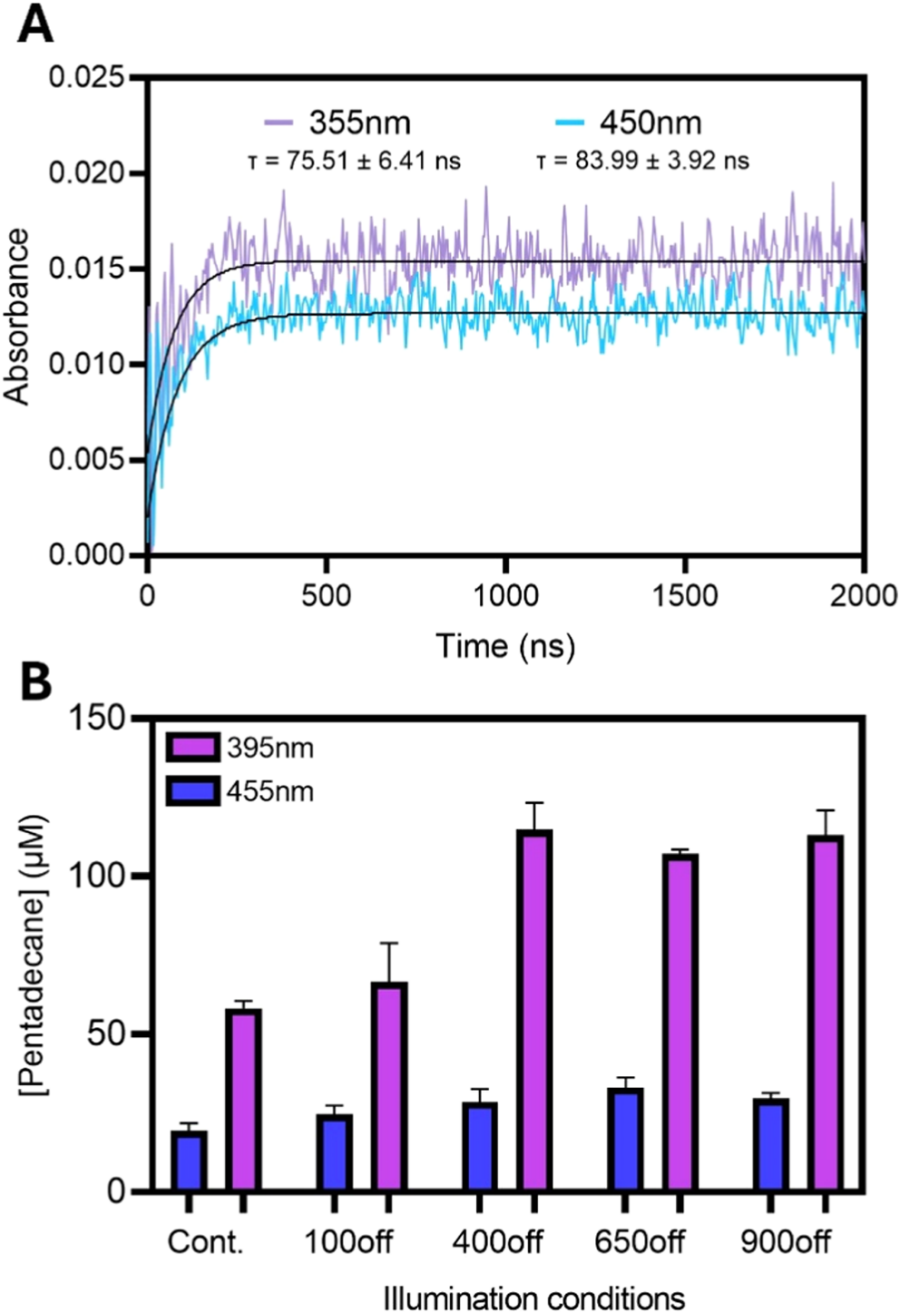
Mechanistic insights into the FAP catalytic cycle upon illumination with violet and blue light. (A) Kinetic transients at 515 nm showing the formation of the red-shifted flavin intermediate monitored by time-resolved laser photoexcitation measurements after excitation at with 355 nm (violet) or blue (450 nm) laser pulse. Samples contained 50 μM *Cv*FAP and 300 μM palmitic acid in Tris-HCl buffer +30% DMSO (pH 8). (B) Pentadecane production by *Cv*FAP using continuous (Cont.) or pulsed light using 455 or 395 nm LEDs. The pulse width was always 100 ms, and the frequency of pulsing is expressed as the ‘off’ time in ms. For continuous light delivery, the assay was run for 1 min at 100 μmol photons m^–2^ s^–1^, and subsequent pulsing assays were performed for the length of time that resulted in equal total photon delivery. Assays were performed at 25 °C in 70 mM Tris-HCl + 30% DMSO (pH 8), with 3 μM FAP and 500 μM palmitic acid.

A mechanistic rationale for the increase in *Cv*FAP activity with violet light was investigated. Initially, we considered the possibility that violet light may initiate a different photocatalytic cycle to that observed previously with blue light.^5,6^ In the case of blue light illumination, it has been shown that excited state electron transfer to the FAD cofactor leads to decarboxylation of the substrate and formation of a novel red-shifted FAD intermediate (FAD_RS_) with a time constant of ∼100 ns.^6^ We measured whether the same FAD_RS_ species is formed upon illumination with violet light using time-resolved laser photoexcitation measurements (Figure 5A). A similar increase in absorbance at 515 nm, indicative of the formation of FAD_RS_, occurred with a time constant of τ = 75–84 ns upon excitation with both violet and blue laser pulses. The production of FAD_RS_ at similar time scales suggests the same light-driven catalytic cycle is observed upon illumination with both wavelengths of light, therefore the occurrence of a different, more productive cycle upon illumination with violet light is unlikely.

Second, we explored the possibility that the enhancement in catalytic efficiency with violet light could be caused by excitation of FAD intermediates from the previous catalytic cycle. Such a mechanism would lead to a more favorable secondary photocycle that results in higher levels of photocatalysis. In such a scenario, the increase in catalytic activity with violet light would only be observed upon continuous illumination of the sample where excitation of transient FAD species is possible, since upon cessation of illumination, FAD will quench rapidly to the ground state. Hence, this hypothesis was investigated by measuring the amount of product that is formed upon continuous illumination compared to the same number of photons delivered in a pulsed manner. In contrast to a reduction in the level of enhanced catalytic activity with violet light that might be expected upon excitation of any transient FAD species, we observed an increase in the product yield when the light is pulsed. A similar effect is observed for blue light, which again indicates that violet and blue light illumination lead to the same reaction pathway. Moreover, the amount of product increases further when the length of time between light pulses is increased and reaches a plateau when the dark period is longer than 400 ms (Figure 5B). It is possible that continuous illumination results in prolonged excitation of nonreactive FAD species (e.g., free or product-bound enzyme), which leads to higher levels of photoinactivation than is observed with pulsed light. By increasing the period between pulses (>400 ms), it is likely that there is sufficient time to allow the previous FAP catalytic cycle to be completed and for a new substrate to rebind (i.e., a catalytic-ready state) prior to the next light pulse.

**Figure 6:**
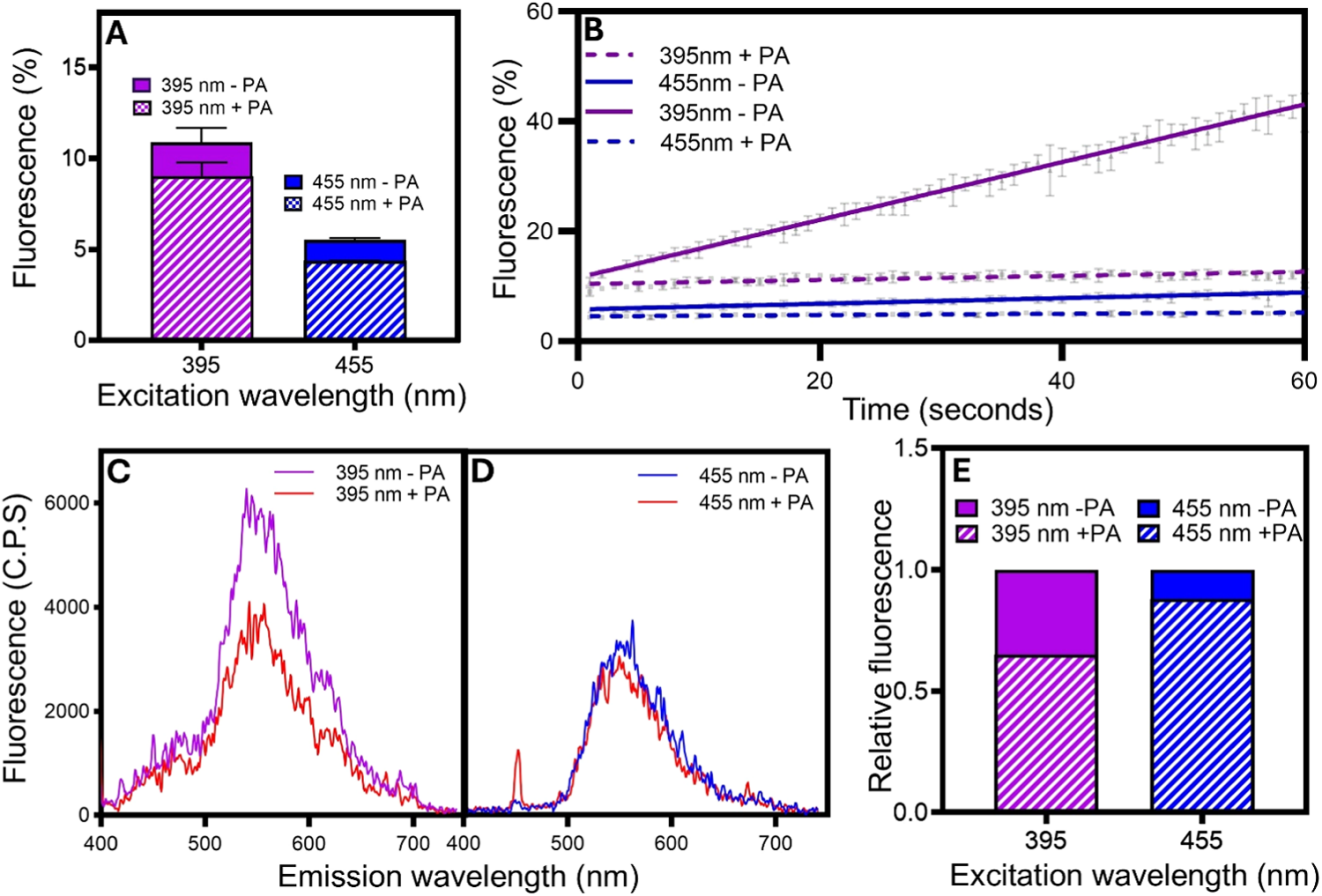
Determination of the level of ^1^FAD* state by fluorescence emission measurements upon illumination with violet and blue light. (AB) Fluorescence of 10 μM *Cv*FAP with and without 500 μM palmitic acid (PA) following pulses of illumination with 455 and 395 nm LEDs at 100 μmol photons m^–2^ s^–1^. Pulses were 100 ms in width, with a period of 900 ms between pulses (one pulse per second). Fluorescence is expressed as a percentage of the total fluorescence emitted from FMN at the same concentration and light intensity. Fluorescence from the first pulse is shown in (A) and fluorescence for subsequent pulses over 1 min is shown in (B). (C, D) Fluorescence emission spectra of 25 μM *Cv*FAP with and without 500 μM palmitic acid upon excitation of a laser pulse at 395 nm (C) or 455 nm (D). (E) The proportion of fluorescence from laser emission spectra in the presence of substrate normalized against fluorescence in the absence of substrate. All experiments were performed at 25 °C in 70 mM Tris-HCl buffer +30% DMSO (pH 8). The concentration of palmitic acid was 500 μM in all cases.

As it is unlikely that the enhancement in catalytic efficiency of FAP upon violet light illumination is caused by either a different catalytic mechanism or by excitation of transient FAD species we explored the relative population of excited singlet state FAD (^1^FAD*) formed upon violet and blue light illumination. Fluorescence emission was used as a reporter for the formation of ^1^FAD* following illumination of *Cv*FAP with identical intensities of 100 ms pulses of violet or blue light (Figure 6A,B). Initially, illumination with violet light exhibited a ∼2-fold increase in fluorescence emission compared to blue light, indicative of a higher population of ^1^FAD*. In the presence of substrate, the emission is quenched to a similar degree upon excitation with both violet and blue light, presumably as the lifetime of the ^1^FAD* state is reduced as a result of photocatalysis. It is known that in the absence of substrate the formation of ^1^FAD* can lead to photoinactivation of the enzyme, most likely via conversion to the triplet state.^31^ In our experiments, the fluorescence emission increases upon exposure to further pulses of light in the absence of substrate, which indicates that the FAD is released from the protein as a result of inactivation. The increase in FAD fluorescent yield is significantly higher with violet light, again suggesting that violet light is much more efficient at forming the ^1^FAD* state. In the presence of the palmitic acid substrate, fluorescence emission was much more stable upon exposure to further pulses of light demonstrating the photoprotective effect of keeping FAP catalytically ‘busy’. In order to provide further evidence for a higher population of ^1^FAD* upon illumination with violet light we measured emission spectra directly upon excitation with a single laser pulse at either 395 or 455 nm (Figure 6C–D). A similar ∼2-fold increase in the fluorescence emission peak at ∼530 nm was observed upon excitation with the violet light laser pulse compared to the blue light pulse. The ratio of the fluorescence emission in the absence of substrate relative to the emission in the presence of substrate provides an estimate for the proportion of ^1^FAD* that is quenched as a result of excited state electron transfer from the substrate. The proportion of ^1^FAD* quenched upon 395 nm laser excitation is ∼35% compared to ∼12% with 455 nm excitation (Figure 6E). Taken together, this implies that the enhancement in catalytic activity upon illumination with violet light compared to blue light is due to a greater population of ^1^FAD* and a higher propensity to abstract an electron from the fatty acid substrate to initiate productive photochemistry.

### Kinetic Modeling of FAP Photochemical Pathways

**Figure 7:**
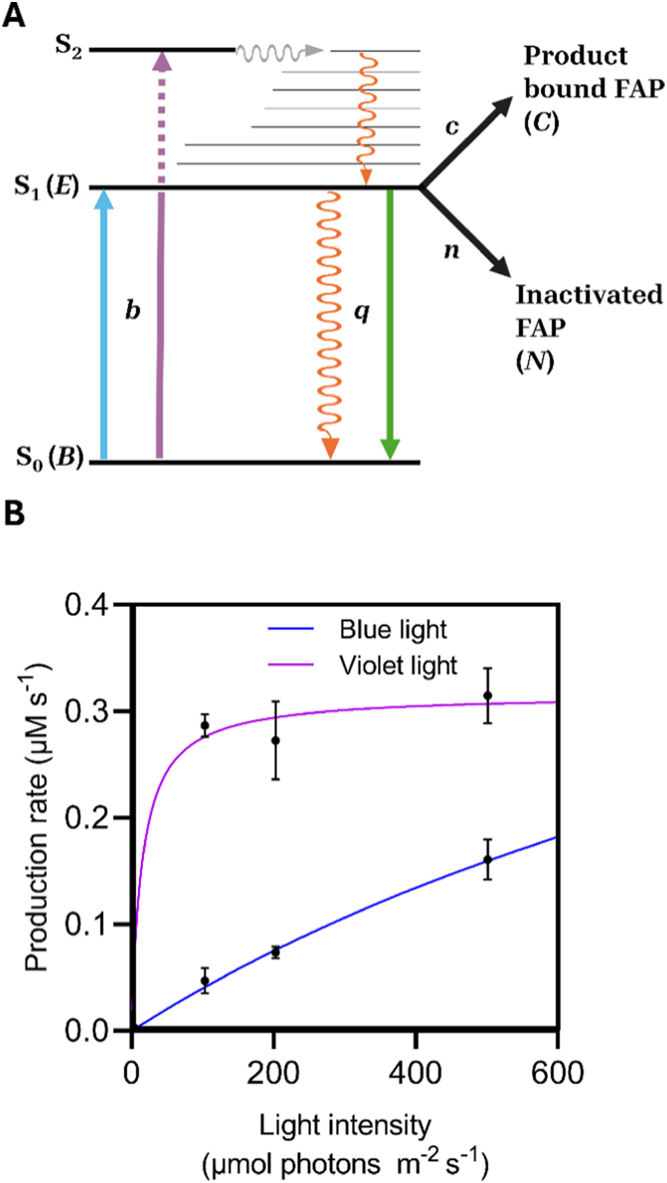
Kinetic processes of FAP photochemistry upon irradiation with blue and violet light. (A) Schematic of the kinetic model used, highlighting the various parameters studied in the present work, in the form of a Jablonski diagram. Blue and violet arrows represent the transition to both the S_1_ and S_2_ excited singlet states (b). Quenching of the excited state (q) is represented as a combination of fluorescence (green arrow) and vibrational relaxation (orange arrow). Internal conversion (gray arrow) and vibrational relaxation (orange arrow) down to the S_1_ state are also shown. From the S_1_ state, FAP can undergo productive photochemistry (c) or inactivation (*n*). (B) Calculated maximum rate (μM s^–1^) against light intensity (μmol photons m^–2^ s^–1^) rate constants for illumination with blue light were estimated from published data^5^ and are consistent with data in Figure 4A. The calculation captures the fast increase and plateau of the reaction rate for violet light and a slower increase of the rate with intensity for 455 nm light.

Modeling of the kinetic processes was employed to further our understanding of how competing photocatalytic and photoinactivation pathways may differ with violet and blue light to explain the observed differences in activity. The model incorporates the three major differences that are observed upon illumination with violet light compared to blue light, namely the higher population of the excited singlet state, increase in photocatalysis and higher levels of photoinactivation (Figure 7A). The kinetics of catalysis and inactivation are represented for the early stages of the reaction under blue and violet light (assuming an effectively constant substrate concentration). The dynamics are described by excitation to the singlet excited state (with rate coefficient *b*), relaxation to the ground state (rate coefficient *q*), reaction chemistry (rate coefficient *c)* and photoinactivation (rate coefficient *n*). Although the fluorescence emission wavelength maximum is identical with both excitation wavelengths based on our static fluorescence measurements, the population of the excited singlet state (*E*) is approximately 2-fold higher upon excitation with violet light compared to blue light. The population of *E* is controlled by the rates of excitation to the excited state and decay back to the ground state. We can assume that the rate of initial excitation remains constant between wavelengths, so any differences are likely to arise from a slower decay to the ground state with violet light. As this involves a combination of radiative (fluorescence) and nonradiative decay processes we hypothesize that the nonradiative decay pathway may be reduced upon excitation with violet light. Such an effect may potentially be caused by excitation to higher energy singlet excited state or via excitation of a different electronic transition with the higher energy violet photon, both of which would result in greater populations of excited state species,^33,34^ as shown in schematic Figure 7A.

In addition to the relaxation pathways back to the ground state, the excited state can also proceed along a catalytic route to the product (represented in the model as a product-bound population *C*) or a nonproductive inactivation pathway, represented as *N*. The fluorescence data presented here indicate that the rates of both processes are also enhanced upon illumination with violet light compared to blue light. By using values of *q*, *c* and *n*, that scale according to the experimental findings presented herein, these three parameters can be used to explain the dependence between light intensity and the observed initial rate upon illumination and the differences between blue and violet light (Figures 7B and S7). Such a description reproduces the increase in activity with violet light under conditions of low photon flux, as well as the faster plateauing for the reaction rate at higher levels of violet light irradiation. When it comes to the yields of the reaction, it follows that each turnover will contain a proportion of product-bound enzyme  and inactivated protein . The observation that reaction yields decrease with violet light at increasing intensities (Figure 4B) implies that an effective photon-dependence exists in the inactivation route described by *n* at least in the case of violet light. Higher energy singlet excited states or different electronic transitions with violet light may result in more efficient electron transfer routes or create localized temperature increases in the active site from vibrational decay pathways that yield higher rates at higher photon currents

## Conclusions

In conclusion, we have investigated the enhancement in the catalytic activity of the photoenzyme, FAP, upon illumination with violet light. A spectrophotometric kinetic assay has allowed us to confirm that there is a ∼6-fold increase in catalytic rate with violet compared to blue light illumination. More detailed spectrophotometric analyses, supported by a kinetic model, have suggested that this increase in activity is due to a greater population of excited state FAD species that can proceed along the productive photochemical route. This study shows that pulses of low-intensity violet light may be the optimal illumination conditions to harness this increased photoactivity while reducing photoinactivation. It will be important to determine whether these illumination conditions will result in similar enhancements in activity as new flavin-based photobiocatalysts continue to be discovered.

## Supporting Information Available

Details of methods, raw data, and control data are shown in figures S1–S7 (PDF)

## Abbreviations

FAP: fatty acid photodecarboxylase
MDH: malate dehydrogenase
PEPC: phosphoenolpyruvate decarboxylase
PEP: phosphoenolpyruvate
FAD: flavin adenine dinucleotide
NAD(H): nicotinamide adenine dinucleotide
