# Supplementary material for: Exploring the Increased Activity of the Blue Light-Dependent Photoenzyme Fatty Acid Photodecarboxylase under Violet Light
Source: ACS Catal. 2025 Mar 31;15(8):6088–97. doi: 10.1021/acscatal.4c07757 (PMC12012757; doi:10.1021/acscatal.4c07757)
Supplement: Supplementary file 1 — cs4c07757_si_001.pdf [file cs4c07757_si_001.pdf]

Supplementary information

Exploring the increased activity of the blue light-dependent photoenzyme fatty acid photodecarboxylase under violet light

Harry J. Spacey, Daniel Healy, Jason M. D. Kalapothakis, Junfeng Ma, Michiyo Sakuma, Perdita E. Barran, Derren J. Heyes* and Nigel S. Scrutton*.

Manchester Institute of Biotechnology, University of Manchester, 131 Princess Street, Manchester M1 7DN, UK

Methods

Table S1. Components of the enzyme-coupled assay.

Component	Stock concentration	Final concentration
Tris-HCl (pH 8)	100 mM Tris<br>100mM NaCl	70 mM Tris<br>70 mM NaCl
DMSO	100%	30% v/v
PEPC	140 U/ml	10 U/ml
MDH	600 U/ml	60 U/ml
$MgSO_4$	250 mM	10 mM
PEP	250 mM	6.5 mM
NADH	10 mM	200 µM

Kinetic modelling of FAP photocatalysis and photoinactivation.

Kinetic models with competing photocatalysis and photoinactivation enable us to understand those underlying processes both qualitatively and quantitatively. In the first instance a model of the irradiated substrate-bound protein was used according to **(Scheme 1)**:

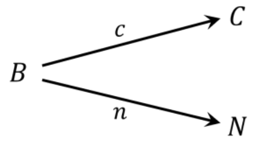

Scheme 1.

Where B is the density of bound protein, C and N being the product-bound protein resulting from catalysis and the inactive fraction of the protein, with c and n being the catalysis and inactivation rate coefficients respectively, show us that the competition between these processes suppresses them compared to what they would have been in the absence of the other one:

$$\frac{dB}{dt} = -(c+n)B$$

$$\frac{dC}{dt} = cB$$

$$\frac{dN}{dt} = nB$$

[Eq. S1a]

With solutions:

$$C(t) = \frac{c}{c+n} B_0\left(1 - e^{-(c+n)t}\right)$$
$$N(t) = \frac{n}{c+n} B_0\left(1 - e^{-(c+n)t}\right)$$

[Eq. S1b]

The rate coefficients c and n determine the fraction of substrate-bound protein that proceed down the catalytic pathway or the inactivation pathway, respectively, providing a kinetic reason for the suppression of inactivation in the substrate-bound form. At all times of such a time course the amount of protein that has participated in catalysis $C(t)$ rather than inactivation, $N(t)$, would be given by:

$$\frac{C(t)}{C(t)+N(t)} = \frac{c}{c+n}$$

[Eq. S1c]

In order to include the photoexcitation and quenching of the excited state of the substrate-bound protein to understand the early-stage kinetics of the catalytic cycle with emphasis on catalysis and inactivation we can include the equilibrium between the substrate-bound state B and the excited state of the substrate-bound complex E, shown in **Scheme 2**.

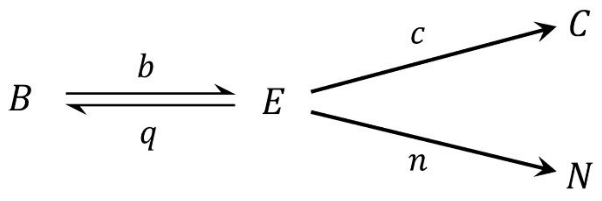

Scheme 2

Corresponding to the following system:

$$\frac{dB}{dt} = -bB + qE$$

$$\frac{dE}{dt} = bB - (q + c + n)E$$

$$\frac{dC}{dt} = cE$$

$$\frac{dN}{dt} = nE$$

[Eq. 2a]

with solution (during irradiation):

$$B(t) = \frac{1}{\sqrt{\mathfrak{D}}}[\zeta_{B+}e^{r_+t} - \zeta_{B-}e^{r_-t}]$$

$$E(t) = \frac{1}{\sqrt{\mathfrak{D}}}[\zeta_{E+}e^{r_+t} - \zeta_{E-}e^{r_-t}]$$

$$C(t) = \frac{c}{\sqrt{\mathfrak{D}}}\left[\frac{1}{r_-}\zeta_{E-}(1 - e^{r_-t}) - \frac{1}{r_+}\zeta_{E+}(1 - e^{r_+t})\right]$$

$$N(t) = \frac{n}{\sqrt{\mathfrak{D}}}\left[\frac{1}{r_-}\zeta_{E-}(1 - e^{r_-t}) - \frac{1}{r_+}\zeta_{E+}(1 - e^{r_+t})\right]$$

$$r_\pm = \frac{1}{2}\big(-(b + q + c + n) \pm \sqrt{\mathfrak{D}}\big)$$

$$\mathfrak{D} = (b + q + c + n)^2 - 4b(c + n)$$

$$\zeta_{B\pm} = (q + c + n + r_+)B_0 + qE_0$$

$$\zeta_{E\pm} = r_\pm E_0 + b(B_0 + E_0)$$

[Eq. 2b]

The maximum rate of production is given by:

$$k_{max} = \frac{1}{\sqrt{\mathfrak{D}}}\left(\frac{{\zeta_{E+}}^2}{\zeta_{E-}} e^{r_+/\sqrt{\mathfrak{D}}} - \zeta_{E+} e^{r_-/\sqrt{\mathfrak{D}}}\right)$$

[Eq. 2c]

Noting that the roots of the characteristic polynomial for this system, $r_\pm$ will be real and *negative* for strictly positive rate coefficients, this model is a function of two appropriately scaled exponential decay functions, and the sum of their exponents would yield the sum of all rate coefficients, $b + q + c + n$, their difference will yield $\mathfrak{D}$ and their product will be $b(c + n)$. As in the system of Equation [1], Equation [2] also obeys the relation $\frac{N}{C} = \frac{n}{c}$, illustrating how both of those branched reaction schemes preserve the kinetic suppression of the slower process; indeed, at late times $C_{t\to\infty} = \frac{c}{c+n}$ and $N_{t\to\infty} = \frac{n}{c+n}$.

Representative kinetic traces for model [2], which interprets the kinetic processes in the main text, are shown in **Fig. S4**

Given that the excitation and relaxation rate coefficients are much higher than the catalysis and inactivation rate coefficients, plotted in **Fig. S4**, upon irradiation the ground state decays rapidly, with concurrent increase in the excited state. The relaxation rate coefficient q is the sum of the coefficients of *all* relaxation processes, including light-scattering, vibrational excitation and light emission, without any loss in generality. Once the excitation-relaxation equilibrium is reached, both the ground and excited state decay as the system proceeds via the electron transfer pathways of productive catalysis and of the “off-pathway” inactivation processes. Similarly, the coefficients c and n are the sum of *all* catalytic and inactivation rate coefficients, respectively.

The kinetics of catalysis (and inactivation, given that $\frac{N}{C} = \frac{n}{c}$) will contain a lag phase at early times, the timescale of which is determined by the timescale of the excitation-relaxation equilibrium. In actual experiments such a lag phase will be in the order of nanoseconds. Once

the ground state and excited states are in equilibrium, the catalytic cycle or inactivation can proceed, increasing the population of product or inactivated protein. The maximum rate is given in Eq. S2c and with a re-interpretation of the rate coefficients can be used to estimate steady-state initial rates.

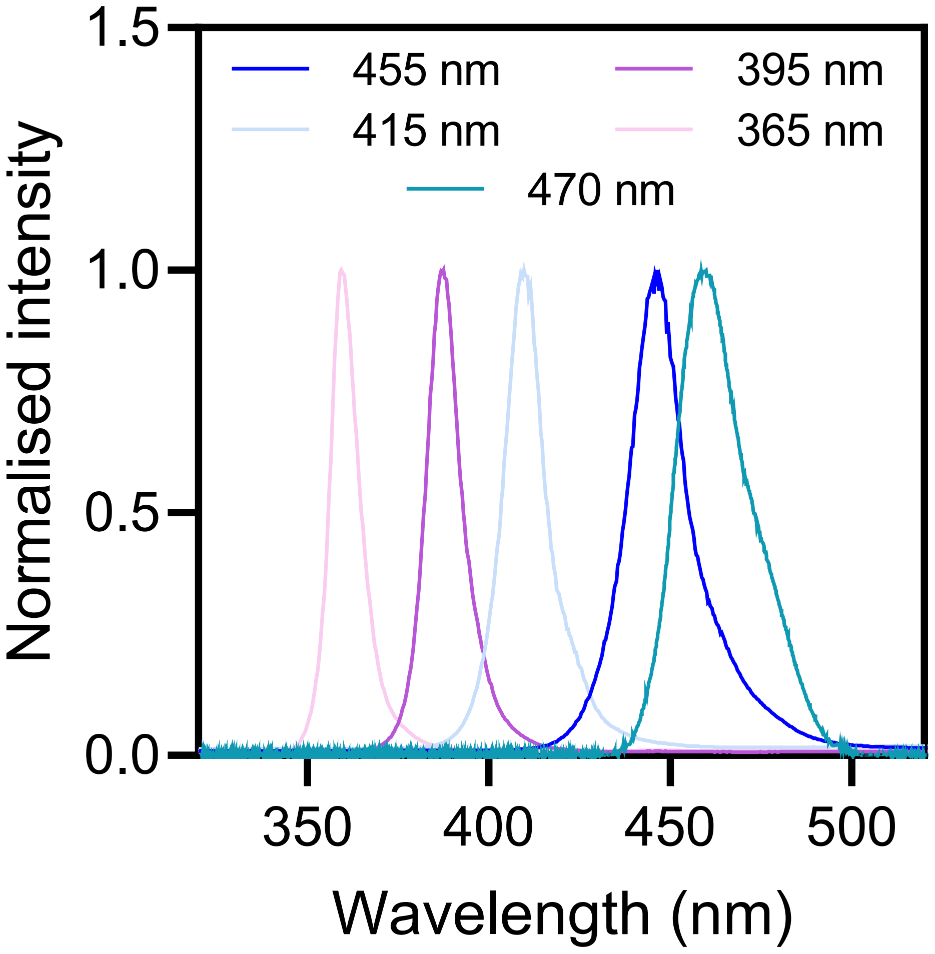

Figure S1. Emission spectra for the LEDs used in the present study.

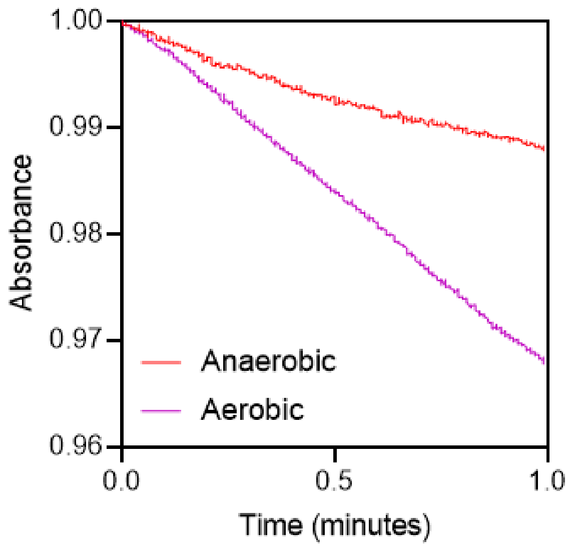

Figure S2. The background rate without additional bicarbonate was measured in aerobic and anaerobic conditions using 1 U/ ml PEC and 6 U/ ml MDH in Tris-HCl pH 8.

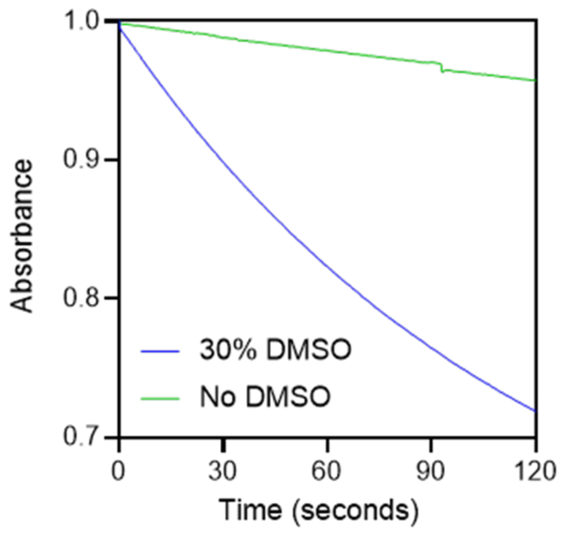

Figure S3. Impact of 30% DMSO on the rate of the enzyme assay. Significantly, there was a large decrease in absorbance at the start of the assays in the absence of light. This was likely not due to FAP turnovers, but a mixing effect from any undissolved palmitic acid causing interference with the spectroscopic reading **(Fig. S3)**. This was an unavoidable feature of using palmitic acid as a substrate and meant that initial rates were taken after this mixing effect had settled (determined by a control performed in the dark).

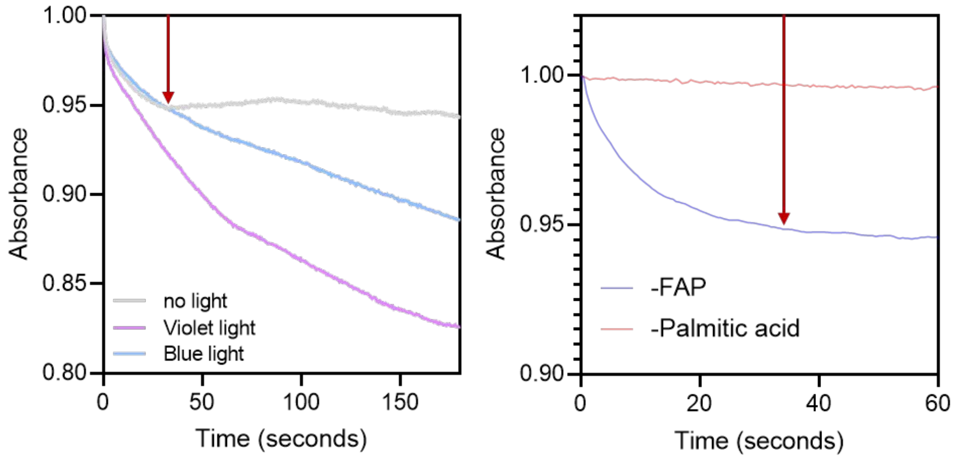

Figure S4. Assay activity in the absence of *Cv*FAP & palmitic acid. The graph on the left shows the trace for *Cv*FAP + 200 µM FAP within the assay in the absence of light (grey) alongside the traces of 455 nm (blue) and 395 nm (violet) under 100 µmol photons m^{-2} s^{-1}. A reduction of absorbance of about 0.05 abs units was measured. Right shows an investigation of the cause of this reduction. When FAP is removed this change in absorbance can still be seen. When palmitic acid is removed instead, the loss of absorbance is no longer present at the start of the assay, suggesting that this reduction is due to undissolved palmitic acid. To compensate for this, initial rates were taken after the first 35 seconds (arrow), to avoid this noise from affecting the readings.

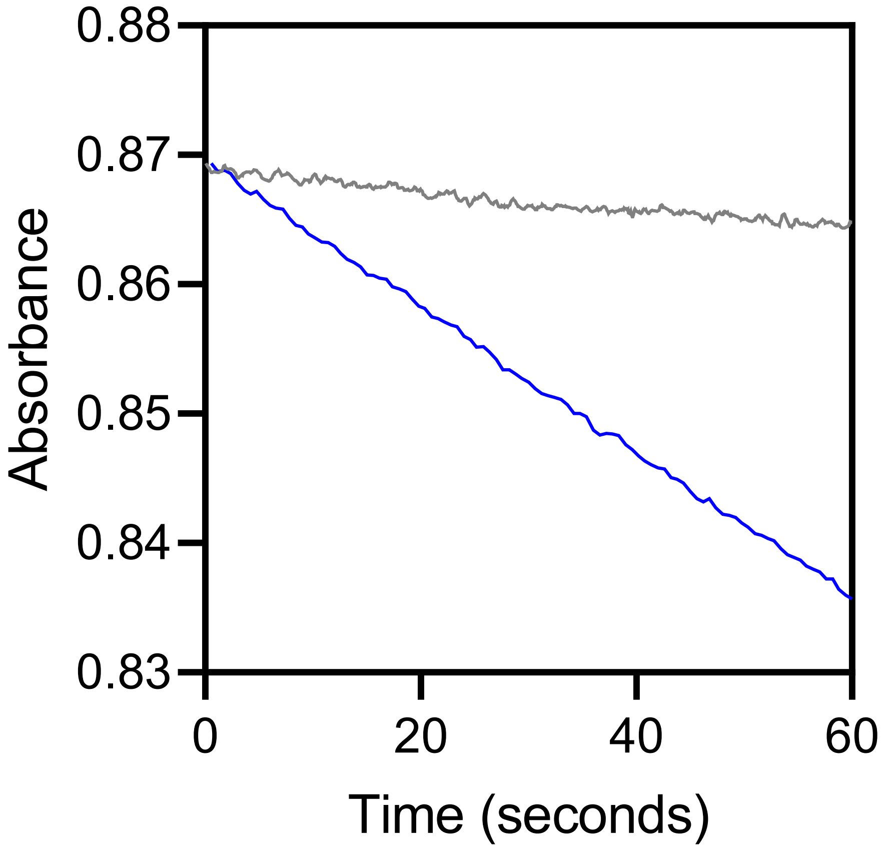

Figure S5. Investigating the effect of the 340 nm measurement lamp. The assay was run with 1 µM FAP + 200 µM palmitic acid in the absence of any external LED light (grey trace) and compared to the lowest light intensity of 455 nm light (blue trace) used in this study (100 µmol photons m^{-2} s^{-1}). A low background rate was observed in the absence of any external light, with ~10-fold lower activity than in the presence of low intensity blue light. The intensity of the measurement lamp was reduced as much as possible during measurements to minimise this effect.

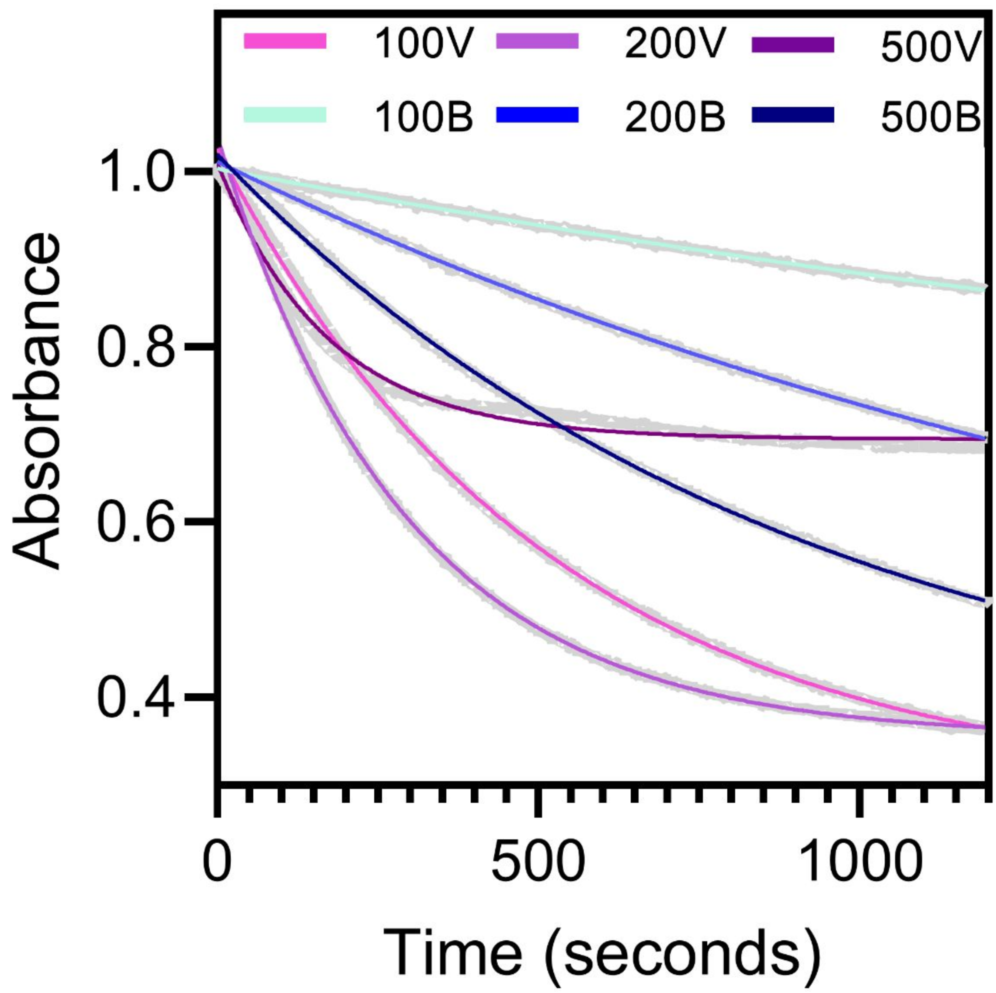

Figure S6. Experimental traces for irradiation of *Cv*FAP over 20 minutes with 395 nm or 455 nm light using the enzyme-coupled assay. Traces (grey) were fitted to a single exponential decay model and inactivation rate constants (k_{inact}), representing the proportion of enzyme that was inactivated per minute, were obtained for each light intensity (R^2= 0.99 for all fits). Light intensity for 395 nm violet (V) and 455 nm blue (B) was measured in µmol photons m^{-2} s^{-1}. Assays were performed at 25 °C in 70 mM Tris-HCl + 30 % DMSO (pH 8), with 2 µM FAP and 200 µM palmitic acid.

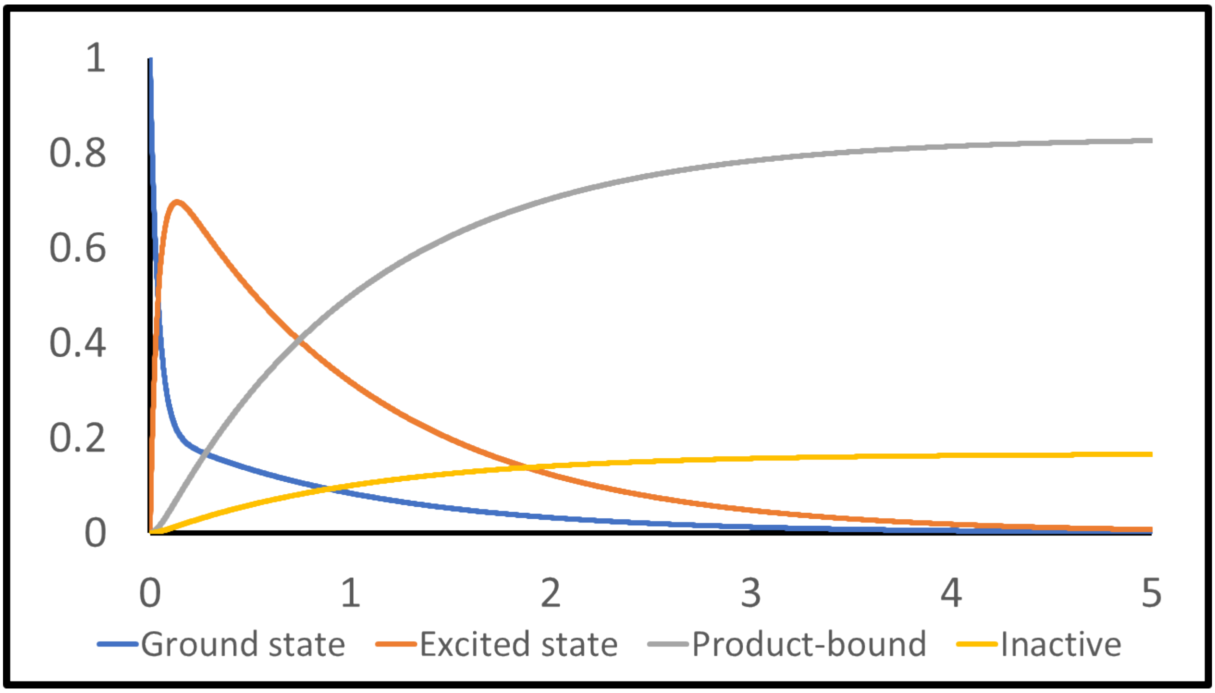

Figure S7. Kinetic traces (concentration vs. time) calculated with Ground state (blue), excited state (orange) productive reaction (grey) and inactivation (yellow) of various pathways under parameters $b = 20, q = 5,\ c = 1, n = 0.2$ s^{-1}. The values of those coefficients are chosen to visually illustrate the patterns in the traces and distinguish on various processes.
